# Down-Regulation of Telomerase Activity and Activation of Caspase-3 Are Responsible for Tanshinone I-Induced Apoptosis in Monocyte Leukemia Cells *in Vitro*

**DOI:** 10.3390/ijms11062267

**Published:** 2010-05-26

**Authors:** Xiao-Dan Liu, Rui-Fang Fan, Yong Zhang, Hong-Zhi Yang, Zhi-Gang Fang, Wei-Bing Guan, Dong-Jun Lin, Ruo-Zhi Xiao, Ren-Wei Huang, He-Qing Huang, Pei-Qing Liu, Jia-Jun Liu

**Affiliations:** 1 Hematological Department & Institute, The Third Affiliated Hospital of Sun Yat-sen University, Guangzhou, Guangdong 510630, China; E-Mails: lenovo381@126.com (X.-D.L.); xfangcn@163.com (R.-F.F.); fzg92@163.com (Z.-G.F.); dongjunlin0168@163.com (D.-J.L.); ruozhi_xiao@yahoo.com (R.-Z.X.); huangrw56@163.com (R.-W.H.); 2 Department of Nuclear Medicine, The Third Affiliated Hospital of Sun Yat-sen University, Guangzhou, Guangdong 510630, China; E-Mail: zy5040@163.com; 3 Department of Traditional Chinese Medicine, The Third Affiliated Hospital of Sun Yat-sen University, Guangzhou, Guangdong 510630, China; E-Mails: hzyang1960@163.com (H.-Z.Y.); gwbing@pulic.guangzhou.gd.cn (W.-B.G.); 4 Lab of Pharmacology and Toxicology, School of Pharmaceutical Science of Sun Yat-Sen University, Guangzhou, Guangdong 510080, China; E-Mails: pql@sysu.edu.cn (P.-Q.L.); huanghq02@163.com (H.-Q.H.)

**Keywords:** Tanshinone I (Tan-I), telomerase, survivin, leukemia

## Abstract

Tanshinone I (Tan-I) is a diterpene quinone extracted from the traditional herbal medicine *Salvia miltiorrhiza Bunge*. Recently, Tan-I has been reported to have anti-tumor effects. In this study, we investigated the growth inhibition and apoptosis inducing effects of Tan-I on three kinds of monocytic leukemia cells (U937, THP-1 and SHI 1). Cell viability was measured by MTT assay. Cell apoptosis was assessed by flow cytometry (FCM) and AnnexinV/PI staining. Reverse transcriptase polymerase chain reaction (RT-PCR) and PCR–enzyme-linked immunosorbent assay (ELISA) were used to detect human telomerase reverse transcriptase (hTERT) expression and telomerase activity before and after apoptosis. The activity of caspase-3 was determined by Caspase colorimetric assay kit and Western blot analysis. Expression of the anti-apoptotic gene Survivin was assayed by Western blot and Real-time RT-PCR using the ABI PRISM 7500 Sequence Detection System. The results revealed that Tan-I could inhibit the growth of these three kinds of leukemia cells and cause apoptosis in a time- and dose-dependent manner. After treatment by Tan-I for 48 h, Western blotting showed cleavage of the caspase-3 zymogen protein with the appearance of its 17-kD subunit, and a 89-kD cleavage product of poly (ADP-ribose) polymerase (PARP), a known substrate of caspase-3, was also found clearly. The expression of hTERT mRNA as well as activity of telomerase were decreased concurrently in a dose-dependent manner. Moreover, Real-time RT-PCR and Western blot revealed a significant down-regulation of Survivin. We therefore conclude that the induction of apoptosis by Tan-I in monocytic leukemia U937 THP-1 and SHI 1 cells is highly correlated with activation of caspase-3 and decreasing of hTERT mRNA expression and telomerase activity as well as down-regulation of Survivin expression. To our knowledge, this is the first report about the effects of Tan-I on monocytic leukemia cells.

## 1. Introduction

**Figure 1:**
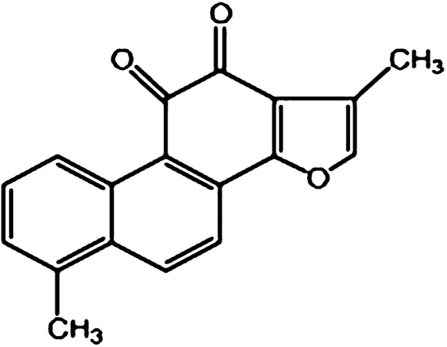
The molecular structure of Tan-I. Reprinted with permission from Chinese Science Press [4].

The use of herbal medicines has become widely accepted as a realistic option for the treatment of malignant disease in recent years [1]. Danshen (Salvia miltiorrhiza Bunge) is one of the widely used Chinese herbal medicines in clinical practice that has been proved to have antimicrobial and antioxidant properties [2,3]. Tanshinone I (Tan-I), as shown in Figure 1, is a diterpene quinine derived from Danshen [4]. Recently, Tan-I has been proved to have anticancer effects in a large variety of cancers such as colon [5], lung [6] and breast cancer [7].

Though Tan-I has been proved to be very effective in treating a variety of malignancies, many of its anti-tumor mechanisms have not been elucidated. Currently, there is no detailed laboratory evidence showing the mechanisms of Tan-I on myeloid leukemia cells. In order to clarify some of its anti-leukemia mechanisms, we investigated the apoptotic effects of various concentrations of Tan-I (10–50 μmol/L) on three kinds of monocytic leukemia cells, U937, THP-1 and SHI 1, *in vitro*.

## 2. Experimental

### 2.1. Main Reagents

Tan-I was kindly provided by Prof. Pei-Qing Liu. The concentration of stock solution is 50 mM with DMSO (Dimethyl sulfoxide, Sigma) and it was diluted to working concentration immediately before use. Reverse transcriptional kit, TRIZOL, Telomerase and Caspase-3 colorimetric assay kits were purchased from R&D systems (Minneapolis, MN, US). Antibodies against Caspase-3, Survivin and poly (ADP-ribose) polymerase (PARP) were purchased from Santa Cruz Biotechnology (Germany). PCR primers used in this study were synthesized by Life Technologies Corporation (Shanghai, China). All other reagents were obtained from Sigma-Aldrich Inc. (St. Louis, MO, USA).

### 2.2. Cell Culture

U937, THP-1 and SHI 1 cells were provided by the central laboratory of Sun Yat-sen university cancer center. Normal peripheral blood monocytes (NPBMs) were isolated from healthy volunteers after obtaining informed consent, by means of Ficoll density gradient centrifugation (specific gravity, 1.077; Shanghai Reagent Factory, Shanghai, China). Cells were cultured in RPMI-1640 medium supplemented with 10% fetal calf serum (FBS), 100U/mL penicillin and l00 μg/mL streptomycin, in a humidified incubator with 5% CO_2_ at 37 °C. All cells were passaged twice weekly and routinely examined for mycoplasma contamination. Cells in logarithmic growth phase were used for further experiments.

### 2.3. Cell Growth Inhibitory Rate (MTT Assay)

Cell inhibitory rate was assayed using the microculture tetrazolium method. Briefly, U937, THP-1 and SHI 1 cells and NPBMs in logarithmic growth phase were collected, and 2 × 10^5^ cells/well were dispensed within 96-well culture plates in 100-mL volumes. Then different concentrations of Tan-I (10, 20, 30, 40 and 50 μmol/L) were put in different wells. Every one of the concentrations above was regarded as one observed group while there was no drug in the control group. Each of the observed groups or control group contained six parallel wells. Culture plates were then incubated for 24, 48, and 72 h, prior to the addition of tetrazolium reagent. MTT working solution was prepared as follows: 5 mg MTT/mL phosphate-buffered saline (PBS) was sterilized by filtering with 0.45-μm filters. To each of the above cultured wells, 20 μL of MTT working solution was added and then incubated continuously for 4 h. All culture medium supernatant was removed from the wells after each of the plates was centrifuged (1000 rpm, 15 min) and replaced with 100 μL of DMSO. Following thorough solubilization, the absorbance (*A* value) of each well was measured using a microculture plate reader at 570 nm. Cell growth inhibitory rate was calculated according to the following formula: Cell growth inhibitory rate = [(*A* value of control group – *A* value of observed group)/(***A*** value of controlled group)] × 100%.

### 2.4. Real-Time RT-PCR Detection of Survivin

After treatment by different concentrations of Tan-I for 48 h, cells were harvested and the total RNA was extracted by using TRIZOL reagent according to the instructions described on the kit. Then the expression of Survivin was detected by Real-time RT-PCR using the ABI PRISM® 7500 Sequence Detection System (Applied Biosystems, Foster City, CA) according to the manufacturer’s instructions. The survivin primers were 5′-AAAGAGCCAAGAACAAAATTGC-3′ (sense) and 5′-GAGAGAGAAGCAGCCACTGTTAC-3′ (antisense), generating a 138 base pair product. For RT-PCR reaction, 250 ng of total RNA were subjected to cDNA synthesis and subsequently amplified during 30 PCR cycles (0.5 sec at 95 °C; 10 sec at 60 °C; 10 sec at 72 °C). Detection limits of the PCR assays were 10^4^ copies for survivin. The threshold cycle (Ct) was calculated by the instrument’s software (7500 Fast System).

### 2.5. Telomerase Activity and hTERT Assay

After the cells were treated by different concentrations of Tan-I for 24, 48, and 72 h, telomerase activity was measured quantitatively by the TRAP–enzyme-linked immunosorbent assay (ELISA) based on the modified telomere repeat amplification protocol (TRAP). The sequences of the primers were: CX 5′-CCCTTACCCTTACCCTTACCCTTA-3′ and TS 5′-AATCCGTCGAGCAGAGTT-3′. Cell extracts were prepared according to TRAP. Telomerase added telomeric repeats TTGGG to the 3′-end of the biotin-labeled synthetic P1-TS primer. Then these elongation products were amplified by PCR using the primers P1-TS and P2-CX to generate PCR products with the telomerase-specific 6-nucleotide increments. Next, an aliquot of the PCR product was denatured and hybridized to a dioxigenin-DIG-labeled, telomeric repeat-specific detection probe. The final product was immobilized to a streptavidin-coated microtiter plate via the biotin-labeled primer and detected with an antibody against dioxigenin (anti-DIG POD) that was conjugated to peroxidase. The probe was visualized by virtue of peroxidase metabolizing TMB to form a colored reaction product. Finally, the absorbance of the samples was measured at 450 nm (with a reference wavelength of approximately 690 nm) using a microtiter plate (ELISA) reader within 30 min after addition of the stop reagent. Absorbance values were reported as the A_450 nm_–A_690 nm_.

RT-PCR was also used to detect hTERT expression. Total RNA was extracted from 1 × 10^6^ cells (after exposure to various concentrations of Tan-I for 48 h) with Trizol RNA kit (Life Technologies, Gaithersburg, Md., USA). To generate hTERT cDNA, a 20-μL reaction containing 1μg of total RNA, 25 mmol/L MgCl_2_, 10 × PCR buffer, 10 mmol/LDntp, 0.1 MDTT, 1 unit/μL of RNase inhibitor, 200 units of murine leukemia virus reverse transcriptase (Life Technologies, Gaithersburg, Md., USA), and 2.5 μmol/L of random hexamers was incubated for 10 min at 65 °C. The samples were then incubated for 60 min at 37 °C, 5 min at 80 °C, and then 5 min at 4 °C. Then 2 μL of reverse transcription product was amplified using the primers yielding a 235-bp fragment. The conditions for PCR were: 94 °C for 2 min, 94 °C for 45 s, 54 °C for 30 s, 72 °C for 60 s, 35 cycles, and 72 °C for 10 min. The positive control was cultured cells with no drugs, and the negative control was inactivated samples (extracted samples were heated at 70 °C for 10 min). PCR products were assayed by 1.5% agarose gel electrophoresis.

### 2.6. Caspase-3 Activity Assay

The activity of caspase-3 was determined by Caspase colorimetric assay kit according to the manufacturer’s protocol. Briefly, 1 × 10^6^ cells treated with different concentrations of Tan-I for 24, 48 or 72 h, were collected, washed with ice-cold PBS and lysed in a lysis buffer. The cell lysates were tested for protease activity using a caspase-specific peptide conjugated with the color reporter molecule p-nitroanaline. The chromophore p-nitroanaline, cleaved by caspases, was quantitated with a spectrophotometer at a wavelength of 405 nm. The caspase enzymatic activities in cell lysates were directly proportional to the color reaction.

### 2.7. Western Blotting Analysis of Caspase-3 and Survivin

After the cells were treated with different concentrations of Tan-I for 48 h, Survivin and caspase-3 was detected by Western blotting. 1 × 10^6^ cells were washed with ice-cold PBS twice and lysed with cell lysis buffer at 4 °C for 30 min. Cell debris was removed by centrifugation at 15,000 × g for 15 min at 4 °C. Equal amounts of proteins were separated by 10% SDS-PAGE and transferred onto nitrocellulose membrane. The membranes were first stained to confirm the uniform transfer of all samples and then incubated in the blocking solution for 2 h at room temperature. The membranes were first incubated with monoclonal antibodies at a dilution of 1:1000 for 2 h, followed by extensive washing with PBS twice and TBST twice. The membranes were then incubated with corresponding horseradish peroxidase-conjugated secondary antibodies (1:1000 dilution) and washed with TBST. β-actin was used as an internal control. The immunoreactive proteins were detected using an ECL Western blotting detection system.

### 2.8. Flow Cytometry (FCM) Detection

After the cells were treated with different concentrations of Tan-I for 48 h, 1 × 10^6^ cells were collected, stained with annexin V and propidium iodide (PI), and then subjected to flow cytometry analysis using FACScan (Becton Dickinson; Mountain View, CA).

### 2.9. Statistical Analysis

All experiments were performed in triplicate. The results were expressed as mean ± SD. For statistical analysis, Student’s t-test were performed using SAS 6.12 software. Statistical significance was accepted at the level of P < 0.05.

## 3. Results

### 3.1. Tan-I Inhibits Monocytic Leukemia Cell Growth

**Figure 2:**
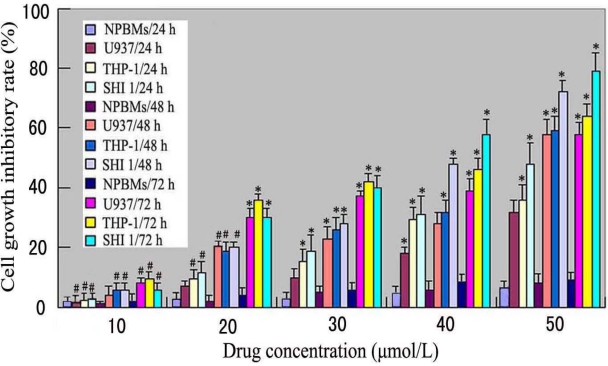
Monocytic leukemia U937, THP-1 and SHI 1 cell growth inhibitory rate caused by Tan-I.

To investigate the growth inhibition effects of Tan-I on leukemia U937, THP-1 and SHI 1 cells, as well as normal peripheral blood monocytes (NPBMs), the cells were treated with various concentrations of Tan-I for 24, 48 and 72 h. As shown in Figure 2, Tan-I (over 20 μmol/L) had significant growth inhibition effects on U937, THP-1 and SHI 1 cells in a dose-and time-dependent manner, while there was no significant cytotoxicity of Tan-I on NPBMs compared to the target leukemia cells. Tan-I with concentrations of 40–50 μmol/L showed much higher growth inhibitory effect than with lower concentrations (p < 0.01).

U937, THP-1 and SHI 1 cells were treated with different concentrations of Tan-I for 24, 48 and 72 h. The cell growth inhibitory rate was determined by MTT assay, as described in “Methods”. Tan-I (over 20 μmol/L) was able to inhibit cell growth in both dose- and time-dependent manner. The cell growth inhibitory rate of Tan-I between 40 μmol/L and 50 μmol/L was much higher than that of 10 μmol/L concentration of Tan-I (*: *p* < 0.01), and there was no significant difference of cell growth inhibitory rate between U937, THP-1 and SHI 1 cells after treatment by 10 or 20 μmol/L Tan-I (# p > 0.05).

### 3.2. Tan-I Inhibits Survivin Expression

**Figure 3:**
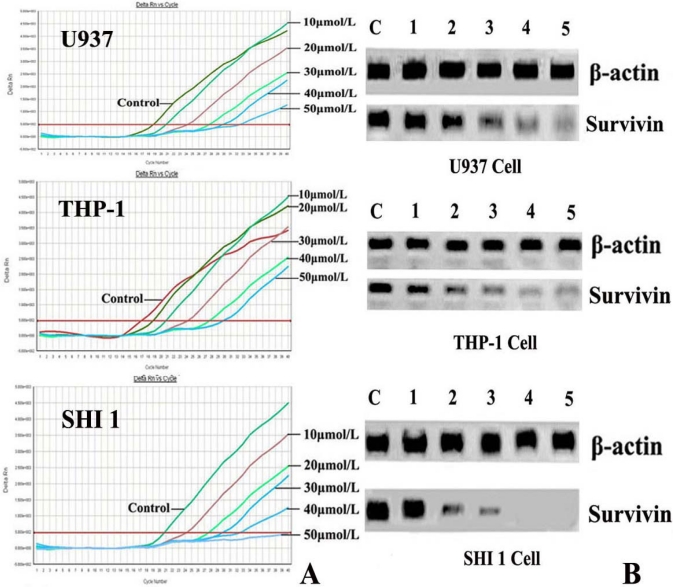
Expression of Survivin in monocytic leukemia U937 cells (top panels), THP-1 cells (middle panels) and SHI 1 cells (bottom panels). (A) Survivin expression was detected by Real-time RT-PCR using the ABI PRISM® 7500 Sequence Detection System after total RNA was extracted. (B) Survivin expression was detected by Western blot analysis. Expression of Survivin was down-regulated in U937, TPH-1 and SHI 1 cells, especially after treatment by 40 or 50 μmol/L Tan-I for 48 h, expression of Survivin was undetectable in SHI 1 cells. β-actin was used as control.

After treatment by different concentrations of Tan-I for 48 h, Survivin expression was first detected by Real-time RT-PCR. As shown in Figure 3A, the expression (copies/mL) of survivin gradually decreased in a dose-dependent manner after Tan-I treatment.

To confirm whether down-regulation of Survivin protein was also involved in Tan-I treated cells, Western blot analysis was also used to detect the expression of Survivin protein. As shown in Figure 3B, Survivin expression was down-regulated dramatically in a dose-dependent manner after Tan-I treatment for 48 h.

### 3.3. Tan-I Inhibits Telomerase Activity and hTERT mRNA Expression

**Figure 4:**
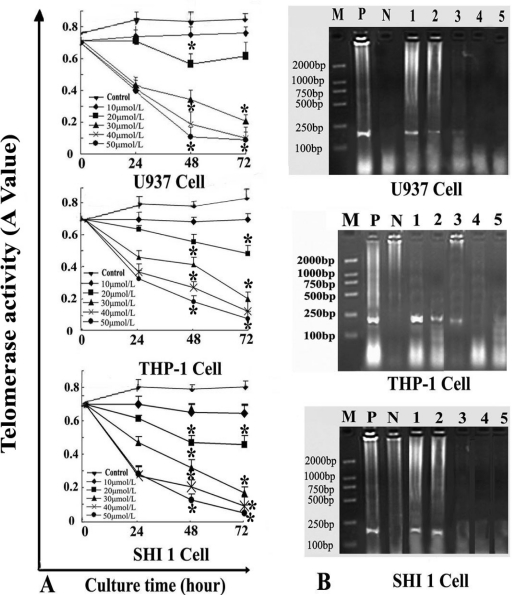
Telomerase activity and hTERT mRNA expression in U937 (top panel), THP-1 (middle) and SHI 1 (bottom panel) cells. (A) Telomerase activity. Telomerase activity decreased in both a time- and dose-dependent manner after Tan-I treatment, and telomerase activity at 40 and 50 μmol/L Tan-I treated cells was down-regulated remarkably compared to that of lower concentrations of Tan-I treated cells and that of the controlled groups (*p<0.01). (B) hTERT mRNA expression of Tan-I treated cells after treatment by different concentrations of Tan-I for 48 h. M: marker: DL 2000. P: positive control, N: negative control. lanes 1–5 were 10, 20, 30, 40 and 50 μmol/L Tan-I.

Telomerase activity was detected in U937, THP-1 and SHI 1 cells. As shown in Figure 4A, telomerase activity of Tan-I treated cells decreased remarkably after Tan-I treatment: the higher the Tan-I concentration, the lower the telomerase activity in the leukemia cells, especially at 72 h—the telomerase activity was undetectable by this method.

hTERT cDNA was also detected by RT-PCR. The full length of hTERT cDNA was 4015 bp, encoding a peptide with 1132 amino acids. The PCR product of should be 235 bp. The agarose gel electrophoresis result showed that the length of the PCR product was consistent with expectations. As shown in Figure 4B, hTERT mRNA expression decreased gradually along with increasing concentrations of Tan-I. No hTERT mRNA expression product was detected when exposed to 40 and 50 μmol/L Tan-I for 48 h.

### 3.4. Tan-I Activates Caspase-3

**Figure 5:**
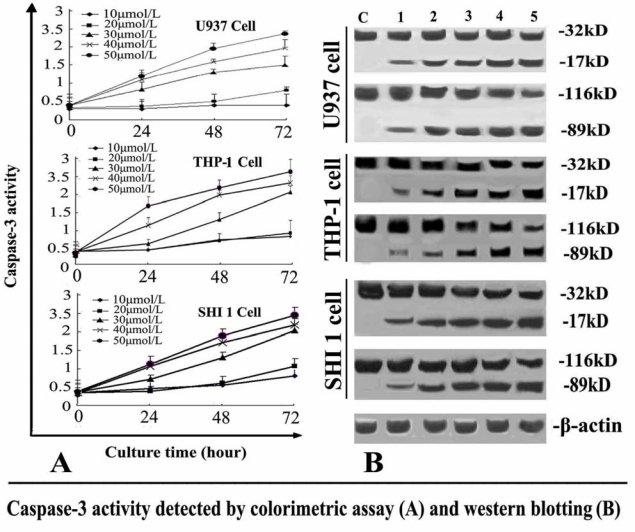
Caspase-3 activity detected by colorimetric assay and Western blot in U937 (top panel), THP-1 (middle) and SHI 1 (bottom panel) cells. (A) Colorimetric assay of caspase-3 activity. Caspase-3 activity was measured as described in the methods. Caspase-3 activity increased remarkably in both a time- and dose-dependent manner; (B) Western blot of caspase-3. Caspase-3 was activated by the loss of the caspase-3 proenzyme (32-kD) and the appearance of its 17-kD subunit (Caspase-3 panels) after the cells were exposed to different concentrations of Tan-I for 48 h. Tan-I treatment also caused a dose-dependent cleavage of PARP, with the appearance of an 89-kD fragment and disappearance of the intact 116-kDa PARP (PARP panels). Bottom panel: Actin control. For all panels, Lane 1, 2, 3, 4 and 5 were 10, 20, 30, 40 and 50 μmol/L Tan-I.

In order to understand the activation of the caspase cascade during Tan-I induced apoptosis in leukemia cells, we first investigated caspase-3 activity after the cells were treated with different concentrations of Tan-I for 24, 48 or 72 h. As shown in Figure 5A, the colorimetric assay revealed that caspase-3 activity increased remarkably in a time-dependent manner.

To estimate the contribution of caspase-3 to Tan-I-induced cell apoptosis, the proteolytic cleavage of pro-caspase-3 was also detected by Western blotting analysis. As shown in Figuge 5B, treatment of the cells with different concentrations of Tan-I for 48 h resulted in the cleavage of the 32-kD caspase-3 zymogen protein and the appearance of its 17-kD subunit. To confirm that Tan-I induced the activation of caspase-3, the cleavage of poly (ADP-ribose) polymerase (PARP) was also examined by Western blotting. As shown in Fig. 5B, Tan-I treatment caused the time-dependent proteolytic cleavage of PARP, with the appearance of an 89-kD fragment and disappearance of the intact 116-kD PARP.

### 3.5. Tan-I Promotes Apoptosis in Leukemia Cells

After the cells were treated with different concentrations of Tan-I for 48 h, cell apoptosis was detected as described in the Methods. Cells were stained with PI and annexin V and then analyzed by FCM. The percentage of apoptotic cells increased in a dose-dependent manner. The experiments were repeated three times and the results are presented as mean ± SD.

**Figure 6:**
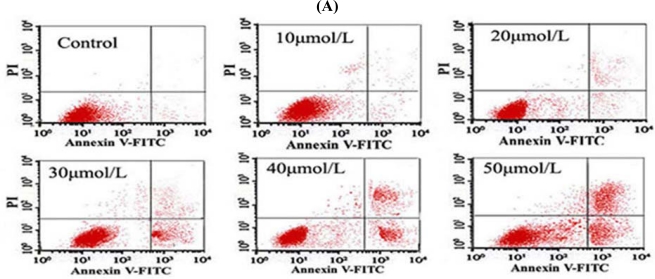

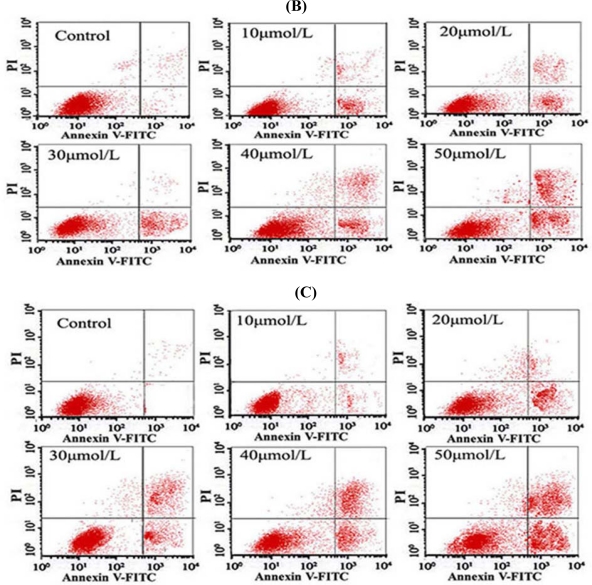
Tan-I induced apoptosis in monocytic leukemia cells. (A) Tan I induced apoptosis in U937 cells; (B) Tan I induced apoptosis in THP-1 cells; (C) Tan I induced apoptosis in SHI 1 cells.

To show the apoptosis inducing effects by Tan-I in *monocytic leukemia* U937 TPH-1 and SHI 1 cells, 1 × 10^6^ cells were harvested after treatment by different concentrations of Tan-I for 48 h, then stained by PI and annexin V and analyzed by FCM. As shown in Figure 6, Tan-I induced apoptosis on leukemia cells in dose-dependent manner. After treatment for 48 h, the apoptotic cells induced by Tan-I treatment were 3.2% (Control), 3.8% (10 μmol/L), 15.4% (20 μmol/L), 17.4% (30 μmol/L), 37.8% (40 μmol/L), 49.7% (50 μmol/L) in U937 cells (Figure 6A), and 3.5% (Control), 4.2% (10 μmol/L), 16.9% (20 μmol/L), 21.4% (30 μmol/L), 42.3% (40 μmol/L), 51.2% (50 μmol/L) in TPH-1 cells (Figure 6B), and 2.5% (Control), 4.3% (10 μmol/L), 17.2% (20 μmol/L), 23.3% (30 μmol/L), 46.8% (40 μmol/L), 54.2% (50 μmol/L) in SHI 1 cells (Figure 6C), respectively.

## 4. Discussion

There are many compounds extracted from *Salvia miltiorrhiza Bunge.* Tan-I is one of its most effective derivatives, which has recently been proved to have activity against a number of cancer cells [5–7]. In this study, we found that Tan-I could inhibit cell growth and induce apoptosis on monocytic leukemia U937, THP-1 and SHI 1 cells remarkably when the concentration of Tan-I was over 20 μmol/L. Caspase-3 activity was upregulated, and Western blot showed cleavage of the caspase-3 zymogen protein (32-kD) with the appearance of its 17-kD subunit when apoptosis occurred. Tan-I treatment also caused a dose-dependent cleavage of PARP, with the appearance of an 89-kD fragment and loss of the intact 116-kD PARP. The hTERT mRNA expression detected by RT-PCR was down-regulated, and telomerase activity gradually decreased along with increasing concentration of Tan-I, especially when the cells were treated with 40 and 50 μmol/L Tan-I for 72 hours. Under these conditions, telomerase activity was undetectable by this method. Furthermore, real-time RT-PCR and Western blot showed that Tan-I treatment caused a dose-dependent decrease in survivin expression. We therefore conclude that the induction of apoptosis by Tan-I in monocytic leukemia cells is highly correlated with activation of caspase-3 and decreasing hTERT mRNA expression and telomerase activity, as well as down-regulation of survivin. These results suggest that Tan-I may be an effective agent for the treatment of leukemia. To our knowledge, this is the first report about the roles of Tan-I on monocytic leukemia cells.

Apoptosis plays an important role in the prevention of the development of cancer. The activation of apoptosis pathways is a key mechanism by which cytotoxic drugs kill tumor cells [8]. Induction of apoptosis has now been considered as an important method for the assessment of the clinical effectiveness of many anti-tumor drugs [9, 10]. Survivin is one of the most important cancer specific proteins. A lot of studies have demonstrated that survivin may play a number of cell functions including inhibition of cell apoptosis, enhancement of tumor cell proliferation by promotion of angiogenesis, and cell-cycle regulation, especially at the mitotic process stage [11]. Recent studies have shown that survivin overexpression is invariably up regulated in a large number of human cancers. Expression of high levels of survivin is closely associated with drug resistance to cancer chemotherapy or radiation therapy, and is linked to poor prognosis in cancer patients [11]. In recent years, the selective induction of apoptosis via down-regulation of survivin expression in tumor cells has been increasingly recognized as a promising approach for cancer therapy [11,12]. Previous studies have demonstrated that survivin is essential for cell cycle progression in leukemia cells, and down-regulation of survivin expression may lead to programmed cell death, suggesting survivin is an appealing new target for the clinical treatment of leukemia [13].

Telomerase is a reverse transcriptase that adds nucleotide repeats to telomeres by using a RNA template, providing karyotype stability and compensating for the loss of DNA [14,15]. Numerous studies have shown that telomerase activity may be detected in over 80% of human tumor cells, and telomerase activity is detectable in almost all cancer cell lines [16,17]. In malignant hematological diseases, high telomerase activity almost always correlates with disease severity, and the activity of telomerase is a very useful index for the diagnosis and clinical staging in hematologic malignancies [18,19]. Recent studies [20] suggest that telomerase inhibitors can promote apoptosis in many hematologic malignancies such as myeloma, and inhibition of telomerase results in telomere shortening, repressed proliferation and altered cell cycle that results in apoptosis in cancer cells [21]. This indicates that anti-telomerase therapy can not only enhance apoptosis in tumor cells, but may also be one of the most important and effective markers for the selection of new anti-tumor drugs [22, 23]. In this study, our results agree with these findings, indicating that down-regulation of telomerase activity may be one of the important mechanisms in Tan I-induced cell growth inhibition and apoptosis in monocytic leukemia cells.

## 5. Conclusion

Tan-I demonstrates apoptosis-inducing effects on monocytic leukemia cells *in vitro* by activation of caspase-3 and decreasing telomerase activity as well as down-regulation of survivin. These results indicate that Tan-I may be an effective anti-leukemia reagents, and that *in vivo* anti-leukemia effects as well as its potential clinical effectiveness need further investigation.
